# Effects of Probiotic Supplementation on Gastrointestinal, Sensory and Core Symptoms in Autism Spectrum Disorders: A Randomized Controlled Trial

**DOI:** 10.3389/fpsyt.2020.550593

**Published:** 2020-09-25

**Authors:** Elisa Santocchi, Letizia Guiducci, Margherita Prosperi, Sara Calderoni, Melania Gaggini, Fabio Apicella, Raffaella Tancredi, Lucia Billeci, Paola Mastromarino, Enzo Grossi, Amalia Gastaldelli, Maria Aurora Morales, Filippo Muratori

**Affiliations:** ^1^ Department of Developmental Neuroscience, IRCCS Stella Maris Foundation, Pisa, Italy; ^2^ Institute of Clinical Physiology, National Research Council, Pisa, Italy; ^3^ Department of Clinical and Experimental Medicine, University of Pisa, Pisa, Italy; ^4^ Department of Public Health and Infectious Diseases, Sapienza University of Rome, Rome, Italy; ^5^ Department of Autism Research, Villa Santa Maria Institute, Tavernerio, Italy

**Keywords:** autism spectrum disorders, probiotics, microbiota-gut-brain axis, gastrointestinal symptoms, inflammatory biomarkers, sensory processing, adaptive functioning

## Abstract

The microbiota-gut-brain axis has been recently recognized as a key modulator of neuropsychiatric health. In this framework, probiotics (recently named “psychobiotics”) may modulate brain activity and function, possibly improving the behavioral profiles of children with Autism Spectrum Disorder (ASD). We evaluated the effects of probiotics on autism in a double-blind randomized, placebo-controlled trial of 85 preschoolers with ASD (mean age, 4.2 years; 84% boys). Participants were randomly assigned to probiotics (De Simone Formulation) (n=42) or placebo (n=43) for six months. Sixty-three (74%) children completed the trial. No differences between groups were detected on the primary outcome measure, the Total Autism Diagnostic Observation Schedule - Calibrated Severity Score (ADOS-CSS). An exploratory secondary analysis on subgroups of children with or without Gastrointestinal Symptoms (GI group, n= 30; NGI group, n=55) revealed in the NGI group treated with probiotics a significant decline in ADOS scores as compared to that in the placebo group, with a mean reduction of 0.81 in Total ADOS CSS and of 1.14 in Social-Affect ADOS CSS over six months. In the GI group treated with probiotics we found greater improvements in some GI symptoms, adaptive functioning, and sensory profiles than in the GI group treated with placebo. These results suggest potentially positive effects of probiotics on core autism symptoms in a subset of ASD children independent of the specific intermediation of the probiotic effect on GI symptoms. Further studies are warranted to replicate and extend these promising findings on a wider population with subsets of ASD patients which share targets of intervention on the microbiota-gut-brain axis.

**Clinical Trial Registration:**

ClinicalTrials.gov, identifier NCT02708901.

## Introduction

Autism Spectrum Disorder (ASD) is characterized by persistent social and communication difficulties along with restricted and repetitive interests and activities (1). The etiopathogenesis of this complex and heterogeneous condition is attributable to early deviation in structural and functional brain development caused by interactions between several genetic and environmental factors, most of which are not yet determined. In recent years, neuroscience research has focused on the role of the microbiota-brain-gut axis in the etiopathogenesis of neurodevelopmental disorders including ASD, thus providing interesting targets for novel psychotropic development (2–8). The gut microbiota can impact brain function, both directly and indirectly, through the production of neurotransmitters, short-chain fatty acids (SCFAs) and key dietary amino acids and their metabolites, as well as through the activation of the immune system that, in turn, could act through inflammatory cytokines and chemokines, such as IL-6 and TNF-α. Moreover, the gut microbiota influence gut barrier permeability, increase the levels of circulating lipopolysaccharide, modulate the levels of brain-derived neurotrophic factor and modify the activity of vagus afferents, enteric nervous system and neuroendocrine pathways such as the hypothalamic-pituitary-adrenal axis. The brain, in turn, modulates gut peristalsis, sensory and secretion function, through the vagus nerve. Gut microbiota perturbations can lead to alterations of all these pathways, thus contributing to the onset or the phenotypic expression of neuropsychiatric and neurodevelopmental disorders (2–8). The possible role of the gut microbiota in ASD has been conceptualized starting from several lines of evidence. First of all, the prevalence of gastrointestinal (GI) symptoms has been found to be higher in ASD subjects compared to typically developing (TD) peers (9–12). Then, several studies showed a significant dysbiosis and a change in the stability, diversity, composition and/or metabolism of the gut microbiota in ASD children compared to TD peers (13, 14), while others reported disrupted intestinal permeability in ASD subjects (15, 16), and evidence of a systemic and intestinal inflammation in ASD [i.e. alterations in circulating cytokine levels (17) and in fecal calprotectin levels (18, 19)]. Studies from ASD-like animal models demonstrated not only that the microbiota are essential for social development (20), but also that restoring the normal components of gut microbiota with probiotics may correct the intestinal permeability defects, altered microbial composition, and ASD-related abnormalities though the reduction of gut production and absorption of toxins (21–23). Probiotics are living microorganisms considered beneficial for human health, generally belonging to Gram-positive taxa, (i.e., *Lactobacillus* and *Bifidobacterium* genus), and recently defined as “psychobiotic” (24), since Dinan et al. (6) have suggested they could be a therapeutic tool useful for altering brain function through their activity in re-establishing the healthy equilibrium of gut microbiota, and modulating tissue neurotransmitter levels. These observations constitute a solid basis for the use of probiotics in ASD, which has been recently addressed also by several preclinical and clinical studies. In particular, an updated review on this topic suggests that probiotic therapy in children with ASD may not only improve the GI dysfunction and the fecal microbiota, but also reduce the severity of ASD symptoms (25). All three studies (26–28) that measured changes in GI function after probiotic supplementation reported a reduction in hard stools, constipation, and diarrhea as well as an increase in formed stools. Therefore, probiotic therapy, despite the variability in species, strains, dosages, and duration among those studies, consistently and beneficially improved the fecal microbiota or urine metabolites. More than half of the investigations also included the assessment of behavioral change measured by different tools, and all of these reported at least nominal (although not always statistically significant) reductions in the severity of ASD symptoms after the probiotic intervention. However, most of the previous studies were affected by several methodological limitations, such as the limited sample size, the strategy of patient enrollment, the criteria for ASD diagnosis, and the study design, mostly being open-label trials or case-control studies (25). Taken together, these findings suggested the need for a randomized, placebo-controlled trial to yield more rigorous results. The current study was a RCT (randomized control trial) evaluating in ASD preschoolers with and without GI symptoms the effects of supplementation with the De Simone Formulation (DSF) on ASD core symptoms, GI symptoms, plasma and fecal inflammatory biomarkers.

## Materials and Methods

### Trial Design

Details of the study design have been previously published (29). The study was a six-months double blind randomized parallel, factorial, efficacy controlled trial with probiotics, with four parallel arms, and an allocation ratio of 1:1. The study protocol was approved by the Pediatric Ethic Committee of Tuscany Region in July 2014 (Approval Number: 126/2014) and registered with Clinicaltrials.gov (NCT02708901). The study was carried out following recognized ethical principles and good clinical practice for clinical trials with food supplements. The protection of individuals was ensured as recommended in the Oviedo Convention and in the Declaration of Helsinki. Written informed consent was obtained from all parents/guardians. The Pediatric Ethic Committee of Tuscany Region assumed a role comparable to a Data Monitoring Committee, requiring the research team to write yearly reports about the progress of the work and reports about any adverse events.

### Participants and Trial Procedures

Participants were enrolled among all the patients assessed in an Italian Tertiary Care Center between November 2015 and February 2018 and screened for eligibility. After recruitment, children were followed up from February 2016 to September 2018. Inclusion criteria were: age-range: 18-72 months; ASD diagnosis according to Diagnostic and Statistical Manual of Mental Disorders-5th Edition (1) (DSM-5) performed by a senior child psychiatrist with specific expertise in clinical evaluation of ASD. Exclusion criteria were: neurological syndromes or focal neurological signs; history of birth asphyxia, severe premature birth or perinatal injuries; epilepsy; significant sensory impairment (e.g., blindness, deafness); diagnosis of not functional GI disorder or Coeliac Disease (e.g. gastroesophageal reflux, food allergies); special diets already underway (i.e. gluten-free diet, casein-free diet, high-protein diet, ketogenic diet); known brain anomalies. After consent was obtained, trial research assessors carried out baseline assessments (T0), which included demographics (age, sex, parental education and employment, family and residential information), medical history, physical examination with anthropometric measurements (weight, height, head circumference), the Autism Diagnostic Interview–Revised (30), primary and secondary outcome measures. Information about pharmacological treatments and food supplements were collected. Due to their possible impact on gut microbiota, information about breastfeeding and food selectivity [assessed using the score at the CBCL item 24 “doesn’t eat well” (11)] were also collected. After baseline assessment, subjects were classified as belonging to the GI group or to the Non-GI (NGI) group through the Gastrointestinal Severity Index [GSI (31)], a composite score designed on a Likert scale to assess signs and symptoms of GI distress reported by parents in the previous two weeks (constipation, diarrhea, average stool consistency, stool smell, flatulence, abdominal pain, unexplained daytime irritability, nighttime awakening, abdominal tenderness). We adopted a GSI cut-off of 4, with at least 3 score points from the first six items of the scale, selected by Adams et al. (13) as more specifically related to GI symptoms and named the 6-GI Severity Index (6-GSI). Children belonging to GI and NGI groups were randomly assigned 1:1 to supplementation with probiotics or with placebo for 6 months, according to a computer generating randomization sequence previously determined which was made in blocks with random sequences of independent block both in the GI and in the NGI groups. The order of interventions varied randomly within each block so that the assignment blocking schedules were unpredictable. The study was double blind till its conclusion for subjects, caregivers and all research investigators. Follow-up assessments at 6 months (T2) after randomization included assessment of outcome measures, adverse events, concomitant treatments, and reasons for dropout. Blood samples were collected at T0 and at T2 by venipuncture in the morning after overnight fasting, rapidly separated by centrifugation for 15 min at 4°C, and plasma samples were stored frozen at −80°C until assay. Fecal samples were collected at home within two days before T0, and T2 and then stored frozen at −80°C until assay. Plasma levels of Leptin, TNF-α, IL- 6, PAI-1 were measured by a specific assay (MILLIPLEX MAP Millipore corporation, Billerica, MA, USA), using an integrated multi-analyte detection platform (high-throughput technology MagPix system, Luminex xMAP technology) with combined Analyst software (MILLIPLEX^®^) for the biomarkers quantification developing new curve fitting algorithms and optimizing mathematical methods to minimize fitting errors. Fecal Calprotectin levels were determined by the quantitative Enzyme-Linked Immunosorbent Assay (ELISA), using a special kit (BÜHLMANN fCAL^®^ ELISA, Buhlmann, Switzerland). All data were stored in paper Case Report Forms and in an electronic database on a secure server with password-controlled access.

### Interventions

The probiotic supplement was DSF, a patented mixture already approved for use in children (marketed as Vivomixx^®^ in EU, Visbiome^®^ in USA). Each packet contained 450 billions of eight probiotic strains: *Streptococcus thermophilus*, *Bifidobacterium breve*, *Bifidobacterium longum*, *Bifidobacterium infantis*, *Lactobacillus acidophilus*, *Lactobacillus plantarum*, Lactobacillus *para-casei*, Lactobacillus *delbrueckii subsp. bulgaricus*. This study protocol required the oral administration of DSF, dissolved directly in the mouth or in a cold, not carbonated liquid at the posology of 2 packets/day in the first month of treatment and 1 packet/day in the following 5 months. The treatment was administered to children at home by the parent(s) or child’s legal guardian. The placebo packaging and organoleptic characteristics were identical to the probiotic ones and contained 4.4 g of maltose and silicon dioxide. The parents/caregivers filled out a weekly food diary in which they reported any suspension in the administration of the experimental treatment and any concomitant drug or food supplement. The suspension of any other intervention effective and recommended by current guidelines in ASD was not required; information on the total number of hours of rehabilitative treatment performed during the study was collected.

### Outcomes

The primary outcome measure was the Total ADOS Calibrated Severity Score (ADOS-CSS) introduced in the Autism Diagnostic Observation Schedule-Second Edition (ADOS-2) (32), for assessing autism severity. The ADOS-2 is a semi-structured assessment considered as the gold standard for the diagnosis of ASD with a demonstrated inter-rater reliability, test-retest reliability, and internal validity. The ADOS-CSS was created to standardize and compare ADOS-2 raw scores across different modules and ages. Calibrated scores are less influenced by the developmental functioning and demographics of the participant than raw totals and are therefore considered the best measure of core features of ASD in pre-school children (33). The ADOS-CSS is useful for comparing assessments across time and identifying trajectories of autism severity for clinical research (34). ADOS-CSS can range on a scale-point from 1 to 10, while raw scores range from 0 to 28, with higher scores indicating greater severity. Secondary measures at T0 and T2 included: Social-Affect (SA) ADOS-CSS, Restricted Repetitive Behaviours (RRB) ADOS-CSS; Vineland Adaptive Behavior Scales-Second Edition (VABS-II) (35) for the evaluation of adaptive functioning; Griffiths Mental Development Scales-Extended Revised (GMDS-ER) (36) for the assessment of developmental level; Total GSI, Total 6-GSI and scores obtained from GSI single items to analyze the severity of GI symptoms; Social Communication Questionnaire (SCQ) Current version (37) for severity of autism symptoms; Sensory Profile, (SP) (38) and Repetitive Behavior Scale-Revised, (RBS-R) (39) to study sensory and repetitive symptoms; Child Behavior Check List 1,5-5 (CBCL 1,5-5) (40) for the evaluation of comorbid psychopathology; Parenting Stress Index (PSI) (41) for the analysis of parental stress; four categories of expressive linguistic level, obtained by combining the score at item A1 of the ADOS-2 (“Total level of spoken language non-echolalic”) with the score at item 30 of the ADI-R (“Overall language level”): 0) language absent or less than 5 words, 1) at least 5 words, 2) sentences of at least 3 words, 3) fluent language. Plasma levels of Leptin, TNF-α, IL- 6, PAI-1, and fecal calprotectin levels were compared at T0 and T2.

### Statistical Analyses

The sample size calculation was based on the primary outcome assumption in the intervention and control groups, the severity level of ASD symptomatology, measured with the ADOS-CSS. In a previous study, the ADOS-CSS decreased in 62% of preschoolers whereas it was the same or worse in 37% of the children after 6 months of “as usual” treatment (42). Sample size calculations were performed using the nQuery advisor 6.2 software. Assuming a response rate of 62% in the placebo group and 90% in the probiotic group, it was calculated that 38 patients per treatment arm would be sufficient to achieve 90% power in detecting a treatment difference based on 1-tail χ2 test at a significance level of 0.05. The main statistical analysis included all participants who had data for the primary endpoint in the group to which they had originally been randomized. Analyses were performed both on the binary outcome measure assumed for sample size calculation (rates of subjects with a decrease in ADOS-CSS vs rates of subjects whereas it was the same or worse) and on continuous outcome measure (changes in mean ADOS-CSS). Quantitative data are presented as mean ± standard deviation (SD). A comparison between different points of time-course was performed by t-student test. The difference between several independent groups was compared by two-way ANOVA. Statistical analysis was performed using Statview 5.0.1 software (SAS Institute, Inc., Cary, NC, USA). A p value <0.05 was considered statistically significant.

## Results

### Baseline Characteristics and Dropouts

**Figure 1:**
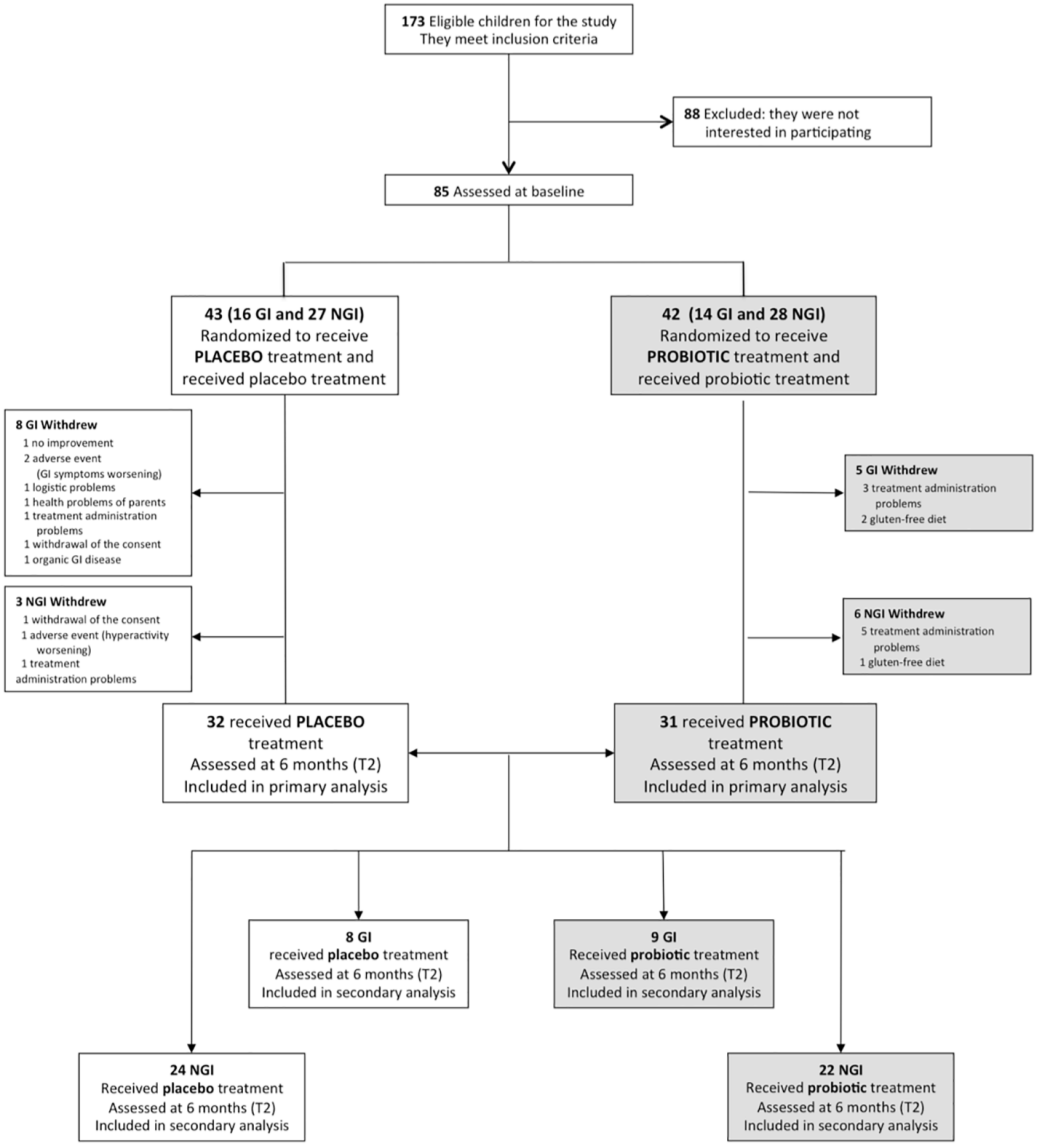
CONSORT flow diagram. GI, Gastrointestinal; NGI, Non-Gastrointestinal.

Of 173 children eligible for the study, 88 declined prior to baseline assessments, before randomization. A total of 85 participants, 55 belonging to the NGI group and 30 belonging to the GI group, were randomized to probiotic supplementation or placebo (42 and 43 respectively) (see **Figure 1**). Baseline demographic and clinical characteristics did not significantly differ between treatment groups (**Table 1**, **Table S2**). Of the 85 participants, all Italian, 71 (84%) were males and the mean age at the recruitment was 4.15 years (SD: 1.08). Sixty-three children completed the trial (placebo: 32, 74.4%; probiotic: 31, 73.8%) with a drop-out rate of 25.9% (22 children: 9 NGI and 13 GI) (see in **Figure 1** reasons for discontinuation). There were no significant differences (p=0.94) in the total number of hours of concomitant rehabilitative treatment over the six-month intervention period in those allocated to placebo (144 ± 86 hours) compared with those allocated to probiotic supplementation (142 ± 114 hours). During the six months of experimental treatment, parents reported (respectively in the probiotic and in the placebo groups): an acute or episodic administration of antibiotics (48.4%, 46.9%), NSAIDs or paracetamol (35.6%, 28.1%), steroids (16.1%, 9,4%), other drugs without effects on GI symptoms (41.9%, 31,3%), a chronic administration of osmotic laxatives (9.7%, 15,6%). None of the enrolled subjects used psychotropic drugs. There were no significant differences in the percentages of children treated with melatonin and vitamins in the probiotic group vs the placebo group (**Table 2**). Baseline characteristics of the 22 children who dropped out at T2 were not significantly different from those of the 63 children who were followed up and included in outcome analysis, except for the GI/NGI ratio, Total GSI 9-items, Total GSI 6-items and RRB ADOS-CSS scores, which were significantly higher in children who dropped out (**Table S1**).

**Table 1:** Baseline characteristics of the sample.

	Groups (n, %)				
Characteristics	Placebo T0 (43, 51)		Probiotics T0 (42, 49)		*p*
**Age, mean (SD), y**		4.13 (1.00)		4.16 (1.17)	ns
**Boys, No. (%)**		37 (86.0)		34 (80.9)	ns
**BMI, SD (Kg/m^2^)**		15.98 (1.62)		15.93 (1.73)	ns
**Food selectivity (%)**		30.2		39.0	ns
**Breastfeeding modalities, (%)**					
**Breastfeeding**		26		32	ns
**Formula feeding**		24		12	ns
**Mixed**		50		56	ns
**ADOS CSS** **^a^** **, mean (SD)**					
Total		7.2 (2.1)		7.0 (1.4)	ns
Social Affect		6.5 (2.2)		6.3 (1.7)	ns
Restricted and repetitive behavior		8.4 (1.3)		8.1 (1.5)	ns
**ADI-R** **^b^** **, n, mean (SD)**	36		36		
Reciprocal social interaction		17.8 (4.8)		18.9 (4.8)	ns
Language and communication		11.7 (2.3)		12.3 (3.5)	ns
Repetitive behaviors and interests		5.3 (1.7)		5.5 (1.7)	ns
Age of onset		4.1 (0.8)		4.1 (0.7)	ns
**SCQ** **^c^**					
**Total score, mean (SD)**		15.8 (5.2)		14.2 (6.6)	ns
**RBS-R** **^d^**					
**Total score, mean (SD)**		21.1 (14.9)		18.5 (12.6)	ns
**DQ** **^e^** **, standardized test, mean (SD)**					
General Quotient, mean (SD)		60.5 (19.1) 25 out of 33		64.6 (16.4) 29 out of 34	ns
Developmental ret. (DQ<70), No. (%)		17 (58.6) 29 out of 43		19 (57.5) 33 out of 41	ns
**VABS II** **^f^** **Composite Score, mean (SD)**		55.4 (17.7)		63.6 (21.0)	ns
**Linguistic Level** **^g^**					
0. No words or < 5 words		26 (60.4)		20 (47.6)	ns
1. At least 5 words		10 (23.2)		11 (26.1)	ns
2. Phrases at least 3 words		6 (13.9)		9 (21.4)	ns
3. Fluent language		1 (2.3)		2 (4.7)	ns
**CBCL** **^h^** **Score, mean (SD)**					
Total Problems		62.9 (10.8)		61.5 (9.9)	ns
**PSI** **^i^** **Score, mean (SD)**					
Total Stress		76.7 (23.1)		69.8 (29.3)	ns
**GI Severity Index** **^j^** **Score, mean (SD)**					
Total 6-GSI		1.8 (1.6)		2.3 (2.2)	ns
Total GSI		3.5 (2.4)		3.8 (3.0)	ns

ADI-R Autism Diagnostic Interview–Revised; ADOS Autism Diagnostic Observation Schedule; CBCL 1.5-5 Child Behavior Checklist 1.5-5; CSS Calibrated Severity Score; D Definite Difference; GI gastrointestinal; GSI Gastrointestinal Severity Index; IQ Intelligence Quotient; No. Number; NGI Non-Gastrointestinal; P Probable Difference; PSI Parental Stress Index; RBS-R Repetitive Behaviors Scale-Revised; SCQ Social Communication Questionnaire; SD Standard Deviation; T Typical Performance; VABS-II Vineland Adaptive Behavior Scales-II.

a Higher scores indicate greater severity (range of possible scores for Total, Social Affect and Restricted and Repetitive Behavior is 1-10).

b Higher scores indicate greater severity (ranges of possible scores: reciprocal social interaction, 0-30; language and communication, 0-26; repetitive behaviors and interests, 0-12; early onset, 0-5).

c Higher scores indicate greater severity (range 0-39) with a threshold of 15 compatible for a relevant impairment of social communication (some studies consider 9 in children younger than four years old).

d Higher scores indicate greater severity of repetitive behaviors (range 0-114).

e Higher scores indicate greater cognitive ability. Scores around 100 indicate normal intelligence; scores below 70 indicate a developmental delay.

f Higher scores indicate greater adaptive competences. Scores around 100 indicate normal adaptive capacities; scores below 70 indicate a delay with respect to age.

g The “Overall Level of Non-Echoed Spoken Language” item (A1 score) of the ADOS-2 was used to differentiate non-verbal (those with absent language or less than 5 words) from verbal children.

h Higher scores indicate greater severity; a score of 63 and above is generally considered clinically significant.

i Higher scores indicate greater severity of parental stress index caused both by characteristics of the child and by negative experiences about the parenting role (Total Stress).

j Higher scores indicate greater severity of gastrointestinal symptoms; Total 6-GSI has a range of 0 to 12, Total GSI has a range of 0 to 17.

**Table 2:** Efficacy Measures at Baseline and 6-Months in the Two Treatment Groups.

	Placebo (32)			Probiotics (31)			p ANOVA Pla/Pro T0	p ANOVA Pla/Pro T0-T2
Characteristics	T0	T2	Change T0-T2	T0	T2	Change T0-T2		
**Age, mean (SD), y**	4.09 (0.97)	4.62 (0.98)	0.52	4.29 (1.22)	4.82 (1.23)	0.53	ns	ns
**Boys, No. (%)**	27 (84.4)	27 (84.4)	n. a.	24 (77.4)	24 (77.4)	n. a.	ns	ns
**BMI m (DS)**	16.06 (1.73)	15.91 (1.76)	-0.15	15.95 (1.93)	16.01 (2.18)	0.06	ns	ns
**Melatonin supplementation n (%)**			2 (6.25)			3 (9.7)		ns
**Vitamin supplementation**			3 (9.4)			7 (22.6)		ns
**ADOS CSS** **^a^**								
Total	6.97 (1.91)	7.00 (1.80)	0.03	6.84 (1.39)	6.19 (1.56)	-0.65	ns	ns
Social Affect	6.41(2.21)	6.09 (1.82)	-0.31	6.26 (1.79)	5.35 (1.56)	-0.90	ns	ns
Restricted and repetitive behavior	8.22(1.31)	8.53 (1.34)	0.31	7.94 (1.57)	8.23 (1.45)	0.29	ns	ns
**SCQ** **^b^**	16.06(5.54)	13.90 (6.19)	-2.16	12.83 (6.68)	11.97 (6.71)	-0.87	0.042	ns
**RBS-R** **^c^**	22.31 (15.47)	19.13 (12.10)	-3.18	18.32 (13.17)	14.37 (8.01)	-3.96	ns	ns
**DQ** **^d^** **, standardized test**								
General Quotient	62.29 (20.12)	61.14 (20.13)	-1.15	65.91 (18.06)	69.27 (20.09)	3.36	ns	ns
**VABS II** **^e^**								
Composite Score	57.00 (16.74)	59.72 (16.38)	2.72	63.87 (22.12)	67.39 (22.29)	3.52	ns	ns
**CBCL** **^f^**								
Total Problems	62.84 (10.97)	57.30 (9.05)	-5.54	60.94 (9.94)	57.80 (7.92)	-3.14	ns	ns
**PSI** **^g^**								
Total Stress	74.76 (24.98)	61.03 (32.58)	-13.72	70.03 (29.63)	66.62 (31.15)	-3.41	ns	ns
**GI Severity Index** **^h^**								
Total 6-GSI	1.38 (1.45)	1.29 (1.19)	-0.08	2.06 (2.14)	1.23 (1.48)	-0.83	ns	ns
Total GSI	2.91 (2.19)	2.16 (1.57)	-0.74	3.61 (2.92)	2.53 (2.19)	-1.08	ns	ns
**Linguistic Level** **^i^**	**Pla T0-T2 (%)**			**Pro T0-T2 (%)**				
	↓	**=**	↑	↓	**=**	↑		
	0	87.50	12.50	9.68	70.97	19.35	ns	ns

ADI-R, Autism Diagnostic Interview–Revised; ADOS, Autism Diagnostic Observation Schedule; CBCL, 1.5-5 Child Behavior Checklist 1.5-5; CSS, Calibrated Severity Score; D, Definite Difference; GI, gastrointestinal; GSI, Gastrointestinal Severity Index; IQ, Intelligence Quotient; No., Number; NGI, Non-Gastrointestinal; Pla, Placebo; Pro, Probiotics; P, Probable Difference; PSI, Parental Stress Index; RBS-R, Repetitive Behaviors Scale-Revised; SCQ, Social Communication Questionnaire; SD, Standard Deviation; T, Typical Performance; VABS-II, Vineland Adaptive Behavior Scales-II. is to be understood as worsened compared to the previous evaluation, **=** is to be understood as unchanged from the previous evaluation, ↑ is to be understood as improved compared to the previous evaluation. Means, and standard deviations are reported.

a Higher scores indicate greater severity (range of possible scores for Total, Social Affect and Restricted and Repetitive Behavior is 1-10).

b Higher scores indicate greater severity (range 0-39) with a threshold of 15 compatible for a relevant impairment of social communication (some studies consider 9 in children younger than four years old).

c Higher scores indicate greater severity of repetitive behaviors (range 0-114).

d Higher scores indicate greater cognitive ability. Scores around 100 indicate normal intelligence; scores below 70 indicate a developmental delay.

e Higher scores indicate greater adaptive competences. Scores around 100 indicate normal adaptive capacities; scores below 70 indicate a delay with respect to age.

f Higher scores indicate greater severity; a score of 63 and above is generally considered clinically significant.

g Higher scores indicate greater severity of parental stress index caused both by characteristics of the child and by negative experiences about the parenting role (Total Stress).

h Higher scores indicate greater severity of gastrointestinal symptoms; Total 6-GSI has a range of 0 to 12, Total GSI has a range of 0 to 17.

i The “Overall Level of Non-Echoed Spoken Language” item (A1 score) of the ADOS-2 was used to differentiate non-verbal (those with absent language or less than 5 words) from verbal children.

### Efficacy: Primary Outcome in the Two Treatment Groups

From baseline to T2, the Total ADOS-CSS decreased in 45.2% (14/31, [95%CI, 27.7% to 62.7%]) of children treated with probiotic and in 28.1% (9/32, [95%CI, 12.5% to 43.7%]) of children treated with placebo. This difference was not statistically significant (risk ratio=1.60; risk difference=0.17; P = 0.16). Mean Total ADOS-CSS scores decreased from 6.84 to 6.19 in the probiotic group and increased from 6.97 to 7.00 in the placebo group, with a difference that did not reach statistical significance (Mean change probiotic vs placebo -0.65 vs +0.03 [95%CI, -0.68 to +0.08]; P = 0.08) (**Table 2**).

### Efficacy: Clinical Secondary Outcomes in the Two Treatment Groups

From baseline to T2, the other pre-specified clinical secondary outcomes showed no significant differences in the probiotic vs the placebo group (**Table 2**, **Table S3**).

### Efficacy: Secondary Exploratory Analyses on GI and NGI Parallel Arms

One of the original aims of this study was to evaluate the effects of probiotics on ASD core symptoms, GI symptoms, and plasma and fecal inflammatory biomarkers in ASD children with and without GI symptoms. For this purpose the randomization was made independently in the GI and NGI groups, to obtain four parallel arms. At the end of recruitment, the sample size of each arm did not reach the target already determined for the whole sample; the GI group, already less numerous, was also affected by a bigger drop-out rate than the NGI one. Therefore, secondary exploratory analyses among subgroups were performed. The four parallel arms were well balanced for the total number of hours of rehabilitative treatments (GI placebo: 175± 91, GI Probiotic 156 ± 68, NGI placebo 134± 84, NGI probiotic 137 ± 129 p>0.05 for all the comparisons).

In the NGI group we found a significant decrease both in the primary outcome measure, Total ADOS-CSS scores (which decreased from 6.72 to 5.91 in the probiotic group and increased from 6.96 to 7.17 in the placebo group; mean change probiotic vs placebo, - 0.81 vs + 0.21 [95%CI, -0.76 to +0.20]; P = 0.026), and in Social-Affect ADOS-CSS (mean change probiotic vs placebo -1.14 vs -0.04 [95%CI, -1.01 to +0.06]; P = 0.027).

In the GI group, statistically significant effects were found in GI symptoms (Total GSI, Total 6-GSI, stool smell and flatulence mean scores), and in adaptive functioning (Receptive Skills, Domestic Skills and Coping Skills VABS-II subscales) for which probiotic therapy was associated with greater improvements than placebo (**Table 3**). In addition, in the GI group a significantly higher proportion of children in the probiotic group than in placebo group showed a normalization of Sensory Profile scores in the Multisensory Processing subscale (p= 0.013): the scores improved in 87% vs 28%, respectively, and got worse in 0% vs 42%, respectively (**Tables S4**, **S5**).

**Table 3:** Efficacy Measures with Significant Changes from Baseline to 6-Months in the Subgroups.

GI SUBJECTS		T0 (baseline)	T2 (after 6 months)	T0-T2	P ANOVA pla/pro T0	P ANOVA pla/pro T0-T2
	**VABS II**					
	**RECEPTIVE** **^a^**					
	PLA	6.75 (3.10)	7.12 (2.53)	0.38 (-1 to 2)		
					ns	0.0104
	PRO	4.78 (3.03)	7.11 (3.14)	2.33 (1 to 6)		
	**DOMESTIC** **^a^**					
	PLA	12.50 (2.27)	13.37 (3.16)	0.87 (-1 to 3)		
					ns	0.047
	PRO	9.44 (5.50)	12.66 (2.74)	3.22 (0 to 14)		
	**COPING SKILLS** **^a^**					
	PLA	11.25 (2.12)	9.75 (4.59)	-1.50 (-8 to 2)		
					ns	0.0115
	PRO	9.11 (4.01)	10.22 (2.17)	1.11 (-1 to 10)		
	**TOTAL 6-GSI** **^b^**					
	PLA	3.50 (0.93)	2.00 (1.53)	-1.75 (-3 to 0)		
					0.009	0.0191
	PRO	5.00 (1.22)	1.67 (1.66)	-3.33 (-6 to 0)		
	**TOTAL GSI** **^c^**					
	PLA	5.75 (1.03)	3.43 (1.81)	-2.28 (-5 to 0)		
					ns	0.0416
	PRO	7.22 (1.99)	2.89 (2.31)	-4.33 (-8 to -2)		
	**GSI, Stool smell** **^d^**					
	PLA	0.25 (0.71)	0.14 (0.38)	-0.15 (-1 to 0)		
					<0.001	<0.001
	PRO	1.88 (0.33)	0.56 (0.88)	-1.32 (-2 to 0)		
	**GSI, Flatulence** **^d^**					
	PLA	0.43 (0.79)	0.86 (0.90)	0.43 (0 to 1)		
					ns	0.0187
	PRO	0.56 (0.88)	0.33 (0.50)	-0.23 (-1 to 0)		
**NGI SUBJECTS**						
	**ADOS**					
	**ADOS CSS social** **^e^**					
	PLA	6.37 (2.30)	6.33 (1.71)	-0.04 (-4 to 4)		
					ns	0.027
	PRO	6.09 (2.00)	4.95 (1.56)	-1.14 (-5 to 2)		
	**ADOS CSS Total** **^e^**					
	PLA	6.96 (1.90)	7.17 (1.79)	0.21 (-4 to 4)		
					ns	0.026
	PRO	6.73 (1.49)	5.91 (1.63)	-0.82 (-4 to 2)		

ADOS, Autism Diagnostic Observation Schedule; D, Definite Difference; GSI, Gastrointestinal Severity Index; No., Number; NGI, Non-Gastrointestinal; Pla, Placebo; Pro, Probiotics; P, Probable Difference; SD, Standard Deviation; T, Typical Performance; VABS-II, Vineland Adaptive Behavior Scales-II. Means, standard deviations, and ranges are reported.

a v-scale: Higher scores indicate greater adaptive competences. Scores around 100 indicate normal adaptive capacities; scores below 70 indicate a delay with respect to age.

b Range of possible scores, 0 to 12; higher scores indicate greater severity.

c Range of possible scores, 0 to 17; higher scores indicate greater severity.

d Range of possible scores, 0 to 2; higher scores indicate greater severity.

e Range of possible scores, 1 to 10; higher scores indicate greater severity.

### Biochemical Secondary Outcomes

No statistically significant changes in plasma biomarkers and in fecal calprotectin levels were found from baseline to T2 in all the subjects who completed the study (**Table 4**).

**Table 4:** Biomarkers at Baseline and after 6-Months in the Two Treatment Groups.

	Placebo T0	Placebo T2	PlaT0-T2	Probiotic T0	Probiotic T2	Pro T0-T2	*p* ANOVA Pla/ProT0	*p* ANOVA Pla/Pro T0-T2
	mean(SD)	mean(SD)		mean(SD)	mean(SD)		
**Plasmatic Biomarkers**								
**IL-6** pg/ml	3.61 (6.18)	3.54 (3.63)	-0.08	3.24 (4.32)	3.33 (2.63)	0.09	ns	ns
**Leptin** pg/ml	1.21 (1.03)	1.32 (1.09)	0.11	1.23 (0.82)	1.13 (0.96)	-0.10	ns	ns
**TNF-α** pg/ml	6.47 (2.79)	6.16 (2.34)	-0.32	5.45 (2.14)	5.82 (2.16)	0.37	ns	ns
**PAI-1** ng/ml	28.79 (23.20)	27.31 (12.54)	-1.48	28.34 (18.15)	32.46 (23.86)	4.11	ns	ns
**Fecal Biomarker**								
**Calprotectin** μgr/gr	128.43 (171.87)	204.61 (438.11)	+76.18	138.12 (196.32)	129.50 (139.67)	-8.62	ns	ns

IL-6, interleukin-6; PAI 1, Plasminogen Activator Inhibitor-1; TNF-α, Tumor Necrosis Factor-alpha; ns, not significant.

### Safety

No serious Adverse Event (AE) was reported. All treatment-emergent AEs were transient and mild in severity. A total of three participants, all treated with placebo, discontinued treatment because of an AE (**Figure 1**), reporting a worsening of GI symptoms (2) and a worsening of hyperactivity (1). Two participants, both treated with probiotic, reported GI symptoms (abdominal pain and diarrhea), during the first ten days of treatment, but these symptoms were transient and both children continued the treatment and completed the trial.

## Discussion

In this double-blind randomized controlled six-months trial completed in 63 children with ASD, the supplementation with probiotic mixture DSF resulted in no statistically significant difference in autism severity as compared with placebo. These results are not consistent with some previous findings of significant improvements in ASD symptoms in response to probiotic administration (26–28). The design of the current study – i.e. the double-blind study protocol and the inclusion of reliable tools to assess outcomes- could explain the differences with those previous investigations. For example, we have used the ADOS-2 (a semi-structured direct observation of the child specifically designed for ASD and administrated by an expert clinician following appropriate training) that is considered a gold standard method of assessment for ASD in both research and clinical practice, even if its capacity to detect changes over time may be questioned (43, 44). Other studies (27, 28) have described significantly superior benefits of probiotics compared to placebo using more subjective instruments as parent-report interviews or questionnaires.

A novel and promising finding of our study is the significant decline in ADOS CSS scores (both Total and Social-Affect scores) in the NGI group treated with probiotics as opposed to those obtained in the placebo group. This result, although deriving from a secondary analysis, is particularly important from a clinical point of view, especially in the light of the abovementioned psychometric properties of the used tool. In fact, a mean reduction of 0.81 in Total ADOS CSS and of 1.14 in Social-Affect ADOS CSS over six months constitutes a clinically significant decrease of ASD symptoms (34). Not all previous trials with probiotics examined their effect taking into consideration the presence/absence of GI symptoms (25). Our result suggests that ASD children with and without GI symptoms could represent two different populations and that probiotics interventions could potentially provide different effects, likely due to distinct microbiota targets. Previous studies have already suggested that differences in microbiome (45, 46) are independent from GI dysfunction, and Luna et al. (45) argued that larger and well-designed studies are still needed to determine whether microbial composition may stratify ASD children beyond the GI symptoms. Within this framework, a positive impact of probiotics on autism severity in children without pre-existing GI symptoms supports the complexity of the microbiota-gut-brain axis warranting further studies on this subgroup of ASD subjects.

As far as GI symptoms, our findings are partially consistent with those reported by some trials (26–28), which showed significant effects of probiotic supplementation in reducing GI symptoms in children with ASD (25). Parracho et al. (26) reported significantly fewer “hard” and more “formed” stools in children treated with probiotic therapy compared with placebo. Shaaban et al. (27) found significant improvement in GI symptoms after three months of probiotic supplementation when measured through 6-GSI, in particular on constipation, stool consistency, flatulence, and abdominal pain. West et al. (28) detected considerable decrease in constipation and diarrhea after probiotic therapy. Our results are also in line with those reported in a recent pilot study performed in 13 ASD children, 3–12 years of age, which showed significant improvement in GI complaints in children treated with DSF compared with children treated with placebo (46).

In the subgroup of children with GI symptoms we found a positive effect of probiotics not only on GI symptoms, but also on adaptive functioning, developmental pathways, and multisensory processing, the latter now reported by the DSM-5 (1) among core symptoms of ASD. The novel finding of a significant improvement in multisensory processing in the GI group could reflect the complex interaction between these two classes of symptoms and their effects on development and adaptive functioning. Specifically, probiotic supplementation, acting on dysbiosis, could reduce distress and enteroception caused by GI symptoms and, consequently, it could ameliorate multisensorial integration process, which in turn is affected by disrupting enteroceptive stimuli determined by dysbiosis. Alternatively, dysbiosis could influence neurotransmitters that play a role in sensory developmental pathways. Recently, difficulties in multisensory processing have been related to the serotoninergic system (47) whose levels are modulated by the gut microbiota. Thus, we could hypothesize that probiotics could ameliorate sensory difficulties thanks to the restoration of the serotonin system that operates also on the reduction of GI symptoms. This result is particularly relevant since positive effects in multisensory processing could have a positive impact on adaptive functioning (48), thus providing a possible explanation for the beneficial effects of probiotics on adaptive functioning we observed in the GI subgroup.

Taken together, these different results on NGI and GI groups of children suggest that the effects of probiotic supplementation in ASD children may be due to distinct mechanisms. The well-known neurobiological heterogeneity of ASD implies that each medication is likely to benefit only a subset within the spectrum of affected children, as suggested by results of pharmacological trials in this population (49, 50). The described positive effect on both GI and NGI children paves the way for the identification of those ASD subjects who can respond to probiotic supplementation beyond the presence of GI symptoms, and even beyond GI inflammatory status. In fact, in the current study, the supplementation with DSF compared with placebo resulted in no significant effects on the levels of plasma and fecal inflammatory biomarkers. In a previous investigation, we have reported that the values of these biomarkers were in the normal range already at baseline (51); thus, we do not confirm the two previous studies (52, 53) reporting some positive effects of probiotics on biomarkers of inflammation, and we could hypothesize that the effect of probiotics on adaptative functioning is not mediated by a reduction in systemic or intestinal inflammation.

Indeed, the exact mechanisms by which probiotics exert potential therapeutic effects are not already completely identified, and they probably go beyond the down-regulation of inflammatory cytokines and refer to other effects on gut barrier permeability, on immunomodulation, and on restoration of altered gut microbiota (54). This is particularly true for the high concentration multi-strain probiotics such as DSF, which has been proven to exert positive effects on balance among different CD4 T-cell subsets and Th17 cell subsets, on the integrity of the gut epithelial barrier, on modulating intraepithelial lymphocytes density and enterocyte apoptosis (55).

The strengths of this study, compared with previous trials of probiotics in ASD, include its duration, rigorous double blinding and simultaneous assessment of several clinical and biochemical outcome measures. Unlike in previous trials, we also controlled for additional rehabilitative treatments in order to ensure that the changes we detected are closely related to the probiotic supplementation. Furthermore, the research protocol administered to the ASD patients seemed very well accepted by parents, children, and staff, with high compliance and adherence to all the procedures. Lastly, our trial confirms the data of previous studies reporting few and transient side effects during probiotic therapy (25), also adding information about the safety of probiotic supplementation in a pediatric population and over a longer period of treatment than previously reported (56).

Several limitations must be noted. Firstly, the large dropout rates, although satisfactory considering the duration of the study, may have affected the trial’s ability to reliably detect significant differences between the two main treatment groups. This seems to have affected particularly the subjects within the GI group, in which almost half of participants dropped out, mostly in the placebo group (as reported in **Figure 1**). We could speculate that parents of these children had more expectations about the efficacy of the probiotic supplementation on GI symptoms than parents of children within the NGI group. For this reason, they could be disappointed when the treatment (or placebo) was not fully effective on GI symptoms of their children, dropping out of the trial without waiting for its possibile positive effects on core and developmental symptoms. Consequently, children who dropped out were substantially comparable to children who completed the trial in all clinical variables, with the exception of higher levels of GI symptoms. This discrepancy between the two groups could impact the study’s ability to detect other possible significant differences in the whole spectrum of GI symptoms. A second limit is that the use of the ADOS-CSS evaluation as an outcome measure in clinical trials has been recently disputed (43), mostly because it lacks sensitivity to detect changes in short time periods. Nevertheless, the field of trials with medication treatments in ASD is still challenged by the lack of objective outcome measures adequately sensitive and specific to change in social symptoms (57); indeed, most studies have used parent-report questionnaires, which lack adequate inter-rater reliability, test-retest reliability, and/or internal validity or are frequently affected by a high placebo effect on parental perception (58). Future research should be addressed to find better outcome measures for detecting changes in ASD core symptoms over time and in clinical trials. Third, the choice of assessing GI symptoms with GSI (a tool not yet validated and providing information based on parent input without added diary) may have affected the reliability of data we collected about GI symptoms. Nevertheless, in a recent literature review (9), comparing different approaches to measurement of GI symptoms (including Autism Treatment Network, Rome criteria, and GSI) in 84 studies on ASD samples, the authors found that no symptom prevalence proportions differed significantly or was associated with the type of questionnaire. Another limitation is related to not having been able to consider possible sex differences in results, since the male skewed sex ratio of the sample (approximately 4:1) did not allow reliable statistical comparisons. Finally, this study did not provide information about microbiota and metabolomic changes during the treatment; future studies need to carry out these analyses in order to search for correlation between brain, clinical improvement and specific composition of microbiota with the ultimate aim of developing precision medicine in ASD.

In conclusion, a six-month probiotic supplementation did not result in statistically significant changes in autism symptoms in the whole sample of ASD preschoolers. Nevertheless, for the first time at our knowledge, we have observed in children without GI symptoms treated with probiotics significant modification of core ASD symptoms measured by the ADOS-CSS scores (specifically Social-Affect domain) that are unrelated to the specific intermediation of the probiotic effect on GI symptoms. As far as children with GI symptoms, the six-month supplementation with DSF showed significant effects, when compared to placebo, in improving not only GI symptoms but also multisensory processing and adaptive functioning.

All these findings could pave the way for further studies on larger subgroups of ASD with the aim of improving precision medicine in ASD.

## Data Availability Statement

The anonymized raw data supporting the conclusions of this article will be made available, without undue reservation, on request to the corresponding author.

## Ethics Statement

The study involved human participants and was reviewed and approved by the Pediatric Ethic Committee of Tuscany Region in July 2014 (Approval Number: 126/2014). Written informed consent to participate in this study was provided by the participants’ parents/legal guardians.

## Author Contributions

ES and LG obtained funding, had full access to all the data in the study, and take responsibility for the integrity of the data and the accuracy of the data analysis. ES, LG, LB, MM, and FM contributed conception and design of the study. ES, LG, MP, LB, SC, MG, FA, RT, PM, EG, and AG contributed acquisition, analysis, or interpretation of data. LG and EG performed the statistical analysis. ES, LG, and MP wrote the first draft of the manuscript. All authors contributed to the article and approved the submitted version.

## Funding

This trial was funded by the Italian Ministry of Health and by Tuscany Region with the grant ‘GR-2011-02348280’. This work was also partially supported by grant from the IRCCS Stella Maris Foundation (Ricerca Corrente, and the “5x1000” voluntary contributions, Italian Ministry of Health to FM, ES, RT, SC, and FA). We are also grateful to Università di Pisa for supporting MP with a research Grant (D.R. n. 33134 29/05/2018). The funders of the study had no role in design and conduct of the study; collection, management, analysis, and interpretation of the data; and preparation, review, or approval of the manuscript or the decision to submit for publication. There was no industry support except for providing probiotic and placebo.

## Conflict of Interest

The authors declare that the research was conducted in the absence of any commercial or financial relationships that could be construed as a potential conflict of interest.
