# Supplementary material for: Effects of Probiotic Supplementation on Gastrointestinal, Sensory and Core Symptoms in Autism Spectrum Disorders: A Randomized Controlled Trial
Source: Front Psychiatry. 2020 Sep 25;11:550593. doi: 10.3389/fpsyt.2020.550593 (PMC7546872; doi:10.3389/fpsyt.2020.550593)
Supplement: Supplementary file 1 [file DataSheet_1.docx]

Sensory Profile Supplementary materials

Table S2. Baseline characteristics

|  |  |  | **Placebo T0 (43)** | |  |  |  | **Probiotics T0 (42)** | |  |  |
| --- | --- | --- | --- | --- | --- | --- | --- | --- | --- | --- | --- |
| **Sensory Profile, n (%)** |  | **T** | **P** | **D** | |  | **T** | **P** | **D** | | **p** |
| Multisensory Processing | 38 | 11 (28.9) | 10 (26.3) | 17 (44.7) | | 32 | 8 (25.0) | 9 (28.1) | 15 (46.9) | | ns |
| Low Registration (SUR) | 43 | 21 (48.8) | 9 (20.9) | 13 (30.2) | | 38 | 15 (39.5) | 10 (26.3) | 13 (34.2) | | ns |
| Sensation Seeking (SS) | 43 | 9 (20.9) | 11 (25.6) | 23 (53.5) | | 38 | 8 (21.1) | 11 (28.9) | 9 (23.7) | | ns |
| Sensory Sensitivity (SOR) | 43 | 13 (30.2) | 13 (30.2) | 17 (39.5) | | 38 | 12 (31.6) | 15 (39.5) | 11 (28.9) | | ns |

Abbreviations (alphabetic order): D definite difference; n number; P probable difference; SOR Sensory Over Responsivity, SS Sensory Sensitivity; SUR Sensory Under Responsivity; T typical performance.

Table S3. Changes at Baseline and 6-Months in the Two Treatment Groups

|  |  | Pla T0-T2 (N=31) | |  | |  | Pro T0-T2 (N=29) | |  | |  |
| --- | --- | --- | --- | --- | --- | --- | --- | --- | --- | --- | --- |
| **Sensory Profile measure, %** | 🡻 got worse | | **=** | | 🡹 improved | 🡻 got worse | | **=** | | 🡹 improved |  |
| Multisensory Processing | 17.86 | | 53.57 | | 28.57 | 16.00 | | 52.00 | | 32.00 | ns |
| Low Registration (SUR) | 19.35 | | 45.16 | | 35.48 | 24.13 | | 44.83 | | 31.03 | ns |
| Sensation Seeking (SS) | 16.13 | | 54.84 | | 29.03 | 17.24 | | 55.17 | | 27.59 | ns |
| Sensory Sensitivity (SOR) | 19.35 | | 64.51 | | 16.13 | 24.13 | | 58.62 | | 17.24 | ns |

Abbreviations (alphabetic order): N number; Pla placebo group; Pro probiotic group; SOR Sensory Over Responsivity, SS Sensory Sensitivity; SUR Sensory Under Responsivity.

Table S4. Changes from Baseline to 6-Months in the NGI Subgroups

|  | PLA T0-T2 | | | |  | PRO T0-T2 | | | | | p | | |
| --- | --- | --- | --- | --- | --- | --- | --- | --- | --- | --- | --- | --- | --- |
| **Low Registration (SUR)** | 24 | =  46 % | Imp  33 % | Worse  21 % | | | 20 | =  45 % | Imp  25 % | Worse  35 % | | NS |  |
| **Sensation Seeking (SS)** | 24 | =  50 % | Imp  29 % | Worse  21 % | | | 20 | =  65 % | Imp  10 % | Worse  25 % | | NS |  |
| **Sensory Sensitivity (SOR)** | 24 | =  66 % | Imp  16 % | Worse  16 % | | | 20 | =  60 % | Imp  10 % | Worse  30 % | | NS |  |
| **Multisensory Processing** | 21 | =  71 % | Imp  28 % | Worse  9 % | | | 17 | =  70 % | Imp  5 % | Worse  23 % | | NS |  |

Abbreviations (alphabetic order): Imp improved; Pla placebo group; Pro probiotic group; Worse got worse; = it remains the same; % percentage; SOR Sensory Over Responsivity, SS Sensory Sensitivity; SUR Sensory Under Responsivity.

Table S5. Changes from Baseline to 6-Months in the GI Subgroups

|  |  | PLA T0-T2 | | |  | PRO T0-T2 | | | P |
| --- | --- | --- | --- | --- | --- | --- | --- | --- | --- |
| **Low Registration (SOR)** | 7 | =  43 % | Imp  43 % | Worse  14 % | 9 | =  44 % | Imp  56 % | Worse  0 % | NS |
| **Sensation Seeking (SS)** | 7 | =  71 % | Imp  29 % | Worse  0 % | 9 | =  33 % | Imp  67 % | Worse  0 % | NS |
| **Sensory Sensitivity (SUR)** | 7 | =  57 % | Imp  15 % | Worse  28 % | 9 | =  55 % | Imp  33 % | Worse  12 % | NS |
| **Multisensory Processing** | 7 | =  28 % | Imp  28 % | Worse  42 % | 8 | =  12 % | Imp  87 % | Worse  0 % | **0.013** |

Abbreviations (alphabetic order): Imp improved; N number; Pla placebo group; Pro probiotic group; Worse got worse; = it remains the same; % percentage; SOR Sensory Over Responsivity, SS Sensory Sensitivity; SUR Sensory Under Responsivity.
