# Supplementary material for: Effects of Probiotic Supplementation on Gastrointestinal, Sensory and Core Symptoms in Autism Spectrum Disorders: A Randomized Controlled Trial
Source: Front Psychiatry. 2020 Sep 25;11:550593. doi: 10.3389/fpsyt.2020.550593 (PMC7546872; doi:10.3389/fpsyt.2020.550593)
Supplement: Supplementary file 2 [file Table_1.docx]

Table S1. Baseline Characteristics of Those Followed up at 6 months Versus Those Who Dropped Out

|  | | **Followed up at 6 months** | | **Dropped out at 6 months** | | ***p*** |
| --- | --- | --- | --- | --- | --- | --- |
| **Characteristics** | | 63 |  | 22 |  |  |
| **Age, mean (SD), y** | | 63 | 4.19 (1.1) | 22 | 4.02 (1.04) | ns |
| **Sex (male), No. (%)** | | 63 | 51 (81) | 22 | 20 (91) | ns |
| **BMI, SD (Kg/m2)** | | 63 |  | 22 |  |  |
|  | |  | 15.99 (1.80) |  | 15.84 (1.20) | ns |
| **Food selectivity (%)** | | 63 |  | 21 |  |  |
|  | |  | 36.5 |  | 28.5 | ns |
| **Breastfeeding modalities, No (%)** | | 62 |  | 21 |  |  |
| **Breastfeeding** | |  | 26 |  | 38 | ns |
| **Formula feeding** | |  | 18 |  | 19 | ns |
| **Mixed** | |  | 56 |  | 43 | ns |
| **ADOS CSS ^a^, No. (%)** | | 63 |  | 22 |  |  |
| **Score, mean (SD)** |  |  |  |  |  |  |
| Total |  | 6.9 (1.7) |  | 7.7 (2.1) | ns |  |
| Social Affect |  | 6.3 (2.0) |  | 6.8 (2.0) | ns |  |
| Restricted and repetitive behavior |  | 8.1 (1.4) |  | 8.9 (1.4) | **.028** |  |
| **ADI-R ^b^, No (%)** | | 63 |  | 9 |  |  |
| **Score, mean (SD)** | |  |  |  |  |  |
| Reciprocal social interaction |  | 18.2 (5.0) |  | 19.8 (3.4) | ns |  |
| Language and communication |  | 11.8 (2.9) |  | 13.8 (3.8) | ns |  |
| Repetitive behaviors and interests |  | 5.5 (1.8) |  | 5.1 (1.8) | ns |  |
| Early onset |  | 4.2 (0.8) |  | 4.0 (0.9) | ns |  |
| **SCQ ^c^, No (%)** | | 62 |  | 21 |  |  |
| **Total score, mean (SD)** | |  | 14.5 (6.3) |  | 16.7 (4.6) | ns |
| **RBS-R ^d^, No (%)** | | 63 |  | 21 |  |  |
| **Total score, mean (SD)** | |  | 20.3 (14.4) |  | 18.4 (12.3) | ns |
| **DQ ^e^, standardized test, No (%)** | | 63 |  | 22 |  |  |
| **Mean (SD)** | |  |  |  |  |  |
| General Quotient, mean (SD) |  | 63.0 (18.7)  49 out of 62 |  | 60.9 (15.9)  13 out of 22 | ns |  |
| Developmental ret. (DQ<70), No. (%) |  | 30 (61)  49 out of 62 |  | 8 (61)  13 out of 22 | ns |  |
| **VABS II ^f^, No (%)** | | 63 |  | 22 |  |  |
| **Score, mean (SD)** | |  |  |  |  |  |
| Composite Score |  | 60.4 (19.7) |  | 57.0 (20.1) | ns |  |
| **Linguistic Level ^g^, No (%)** | | 63 |  | 22 |  |  |
| 0. No words or < 5 words |  | 31 (49) |  | 15 (68) | ns |  |
| 1. At least 5 words |  | 16 (25) |  | 5 (22) |  |  |
| 2. Phrases at least 3 words |  | 13 (20) |  | 2 (9) |  |  |
| 3. Fluent language |  | 3 (4) |  | 0 |  |  |
| **CBCL ^h^, No (%)** | | 63 |  | 21 |  |  |
| **Score, mean (SD)** | |  |  |  |  |  |
| Total Problems | 63 | 61.9 (10.4) | 21 | 63.3 (10.4) | ns |  |
| **PSI ^i^, No (%)** | | 63 |  | 16 |  |  |
| **Score, mean (SD)** | |  |  |  |  |  |
| Total Stress | 60 | 72.3 (27.3) | 14 | 78.1 (22.0) | ns |  |
| **GI Severity Index ^j^, No (%)** | | 63 |  | 22 |  |  |
| **Score, mean (SD)** | |  |  |  |  |  |
| Total 6-GSI |  | 1.7 (1.8) |  | 3.2 (2.1) | **.003** |  |
| Total GSI |  | 3.3 (2.6) |  | 5.0 (2.9) | **.012** |  |
| **NGI subjects, No (%)** |  | 46 (73) |  | 9 (40) | **.007** |  |
| **GI subjects, No (%)** |  | 17 (26) |  | 13 (59) |  |  |

Abbreviations: ADI-R Autism Diagnostic Interview–Revised; ADOS Autism Diagnostic Observation Schedule; CBCL 1.5-5 Child Behavior Checklist 1.5-5; CSS Calibrated Severity Score; D Definite Difference; GI gastrointestinal; GSI Gastrointestinal Severity Index; IQ Intelligence Quotient; No. Number; NGI Non-Gastrointestinal; P Probable Difference; PSI Parental Stress Index; RBS-R Repetitive Behaviors Scale-Revised; SCQ Social Communication Questionnaire; SD Standard Deviation; T Typical Performance; VABS-II Vineland Adaptive Behavior Scales-II.

**^a^** Higher scores indicate greater severity (range of possible scores for Total, Social Affect and Restricted and Repetitive Behavior is 1-10).
**^b^** Higher scores indicate greater severity (ranges of possible scores: reciprocal social interaction, 0-30; language and communication, 0-26; repetitive behaviors and interests, 0-12; early onset, 0-5).
**^c^** Higher scores indicate greater severity (range 0-39) with a threshold of 15 compatible for a relevant impairment of social communication (some studies consider 9 in children younger than four years old).
**^d^** Higher scores indicate greater severity of repetitive behaviors (range 0-114).
**^e^** Higher scores indicate greater cognitive ability. Scores around 100 indicate normal intelligence; scores below 70 indicate a developmental delay.
**^f^** Higher scores indicate greater adaptive competences. Scores around 100 indicate normal adaptive capacities; scores below 70 indicate a delay with respect to age.
**^g^** The “Overall Level of Non-Echoed Spoken Language” item (A1 score) of the ADOS-2 was used to differentiate non-verbal (those with absent language or less than 5 words) from verbal children
**^h^** Higher scores indicate greater severity; a score of 63 and above is generally considered clinically significant.
**^i^** Higher scores indicate greater severity of parental stress index caused both by characteristics of the child and by negative experiences about the parenting role (Total Stress).
**^j^** Higher scores indicate greater severity of gastrointestinal symptoms; Total 6-GSI has a range of 0 to 12, Total GSI has a range of 0 to 17.
